# Exploring van der Waals Cuprate Superconductors Using a Hybrid Microwave Circuit

**DOI:** 10.1021/acs.nanolett.4c05793

**Published:** 2025-01-27

**Authors:** Haolin Jin, Giuseppe Serpico, Yejin Lee, Tommaso Confalone, Christian N. Saggau, Flavia Lo Sardo, Genda Gu, Berit H. Goodge, Edouard Lesne, Domenico Montemurro, Kornelius Nielsch, Nicola Poccia, Uri Vool

**Affiliations:** † Max Planck Institute for Chemical Physics of Solids, 01187 Dresden, Germany; ‡ Institute of Solid State and Material Physics, Technische Universität Dresden, 01062 Dresden, Germany; § Department of Physics, University of Naples Federico II, Via Cintia, 80126 Naples, Italy; ∥ Leibniz Institute for Solid State and Materials Science Dresden (IFW Dresden), 01069 Dresden, Germany; ⊥ Institute of Applied Physics, Technische Universität Dresden, 01062 Dresden, Germany; # DTU Electro, Department of Electrical and Photonics Engineering, Technical University of Denmark, 2800 Kongens Lyngby, Denmark; ¶ Center for Silicon Photonics for Optical Communications (SPOC), Technical University of Denmark, 2800 Kongens Lyngby, Denmark; ○ Institute of Materials Science, Technische Universität Dresden, 01062 Dresden, Germany; △ Condensed Matter Physics and Materials Science Department, Brookhaven National Laboratory, Upton, New York 11973, United States

**Keywords:** van der Waals materials, superconducting microwave resonators, hybrid circuits, two-level-system bath, cuprate superconductors

## Abstract

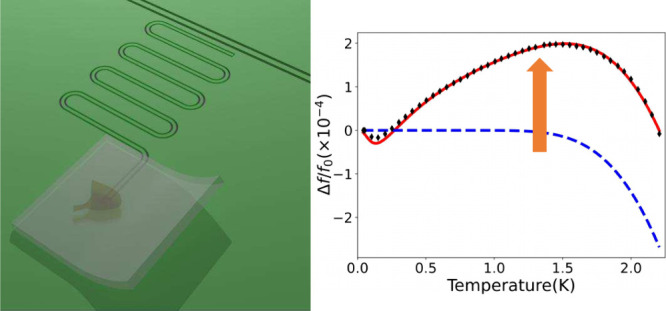

The advent of two-dimensional van der Waals materials is a frontier of condensed matter physics and quantum devices. However, characterizing such materials remains challenging due to the limitations of bulk material techniques, necessitating the development of specialized methods. Here, we investigate the superconducting properties of Bi_2_Sr_2_CaCu_2_O_8+*x*_ flakes by integrating them with a hybrid superconducting microwave resonator. The hybrid resonator is significantly modified by the interaction with the flake while maintaining a high quality factor (3 × 10^4^). We also observe a significant upshift of the resonator frequency with increasing temperature, as well as a positive nonlinearity. These effects originate from a presently unknown microscopic mechanism within the flake, and can be modeled as a two-level system bath interacting with the resonant mode. Our findings open a path for high quality hybrid circuits with van der Waals flakes for exploring novel materials and developing new devices for quantum technology.

Two-dimensional van der Waals (vdW) materials are a rapidly growing field, with new materials exhibiting a wide range of phenomena, including ballistic transport,^1,2^ magnetism,^3−5^ topology^6−8^ and unconventional superconductivity.^9−12^ A key advantage of vdW materials is their versatility in tuning physical properties through in situ electrical gating and the creation of novel material systems by stacking different material layers and varying their relative angle.^13,14^

However, a major challenge arises in the characterization and measurement of vdW flakes because many of the techniques available for exploring bulk materials cannot be directly applied to 2D flakes. Their reduced dimensionality, small size, and delicate nature pose significant challenges, necessitating the development and adaptation of specialized techniques. For instance, the London penetration depth measurement is a common technique to uncover the superconducting gap symmetry,^15,16^ but it requires large, pure crystal samples and is inapplicable for atomically thin and micrometer-sized flakes.

An alternative measurement technique has been recently developed to integrate the materials into a hybrid superconducting resonator.^17−19^ Superconducting resonators are coherent macroscopic devices with high tunability and low-loss operation,^20−23^ making them particularly well-suited for quantum technology^24,25^ as well as sensing applications. Indeed, recently superconducting resonators have been used in combination with vdW materials to investigate their microwave losses,^26^ dielectric properties,^27,28^ kinetic inductance,^29,30^ and coupling to novel Josephson junctions.^31−34^

But the fabrication of a hybrid device composed of a superconducting resonator and a vdW flake presents significant challenges. Superconducting resonators have been meticulously optimized over the years through careful selection of materials and fabrication processes to achieve high coherence. Similarly, the techniques to isolate and preserve pristine vdW flakes have been carefully refined. The interface between these devices can introduce imperfections and compromise both the coherence of the resonator and the structure of the flake. For this reason, the fabrication of hybrid devices requires a dedicated controlled procedure and this is particularly true for complex and sensitive materials. A particularly sensitive but remarkable vdw material is Bi_2_Sr_2_CaCu_2_O_8+*x*_ (BSCCO). BSCCO has garnered significant attention in recent years due to the experimental advances in preserving nearly perfect lattice and superconductivity in the atomically thin limit^10,11^ and for the realization of ultraclean interfaces of twisted vdw heterostructures.^35−37^ This has led to a flourishing of theoretical predictions for new quantum states of matter^38−41^ and further evidence for a dominant d-wave order parameter.^42^

In this work, we explore the microwave properties of an optimally doped vdW BSCCO flake by integrating it into a hybrid superconducting resonator. We utilized a cryogenic stacking technique in a controlled environment, thus ensuring that the pristine structure of the crystal remains unharmed during sample preparation. The resonant mode is strongly modified by the presence of the vdW flake, indicating a strong hybridization, while maintaining a high-quality factor (3 × 10^4^). The hybrid circuit’s microwave response is noticeably different from the bare resonator, showing a substantial increase of the resonance frequency with temperature. This behavior can be modeled as a two-level system (TLS) bath interacting with the resonator. However, the resonator coherence which remains high despite the strong coupling to the TLS bath deviates from the model prediction. Furthermore, the hybrid circuit shows a strong positive nonlinearity, inconsistent with typical resonator nonlinearity due to kinetic inductance. These effects are inherent within the BSCCO flake and possibly correspond to an off-resonant TLS bath, though their exact microscopic origin requires further investigation.

Superconductivity in a BSCCO flake is crucially affected by changes of the spatial configurations of oxygen dopants, which become mobile above −73 °C.^43−45^ Therefore, we employ a cryogenic transfer technique that preserves the spatially correlated superlattice order and freezes oxygen defects in their original positions.^36,37^ We transferred the pre-exfoliated BSCCO flake onto the superconducting resonator using a polydimethylsiloxane (PDMS) polymer. PDMS exhibits increased adhesion at lower temperatures, making it suitable for cryogenic applications. The transfer process was conducted while the sample was cooled to −90 °C, freezing oxygen dopants (Figure 1a). The temperature of the sample stage was then raised to 10 °C (Figure 1b), allowing us to detach the PDMS while minimizing mechanical stress. The 450 nm flake was thus placed at the end of a coplanar resonator made out of 60 nm thick niobium thin film (Figure 1c). To demonstrate the quality of the device at the atomic resolution, we use scanning transmission electron microscopy (STEM) on a cut where the BSCCO flake is held above the niobium in the gap between the center line and the ground plane (Figure 1d). The BSCCO flake maintains perfect crystalline structure with no degradation layer at the interface (Figure 1e). The pristine contact is improved by the amorphous silicon capping layer placed on the niobium, preventing its oxidation (see Supporting Information).

**Figure 1:**
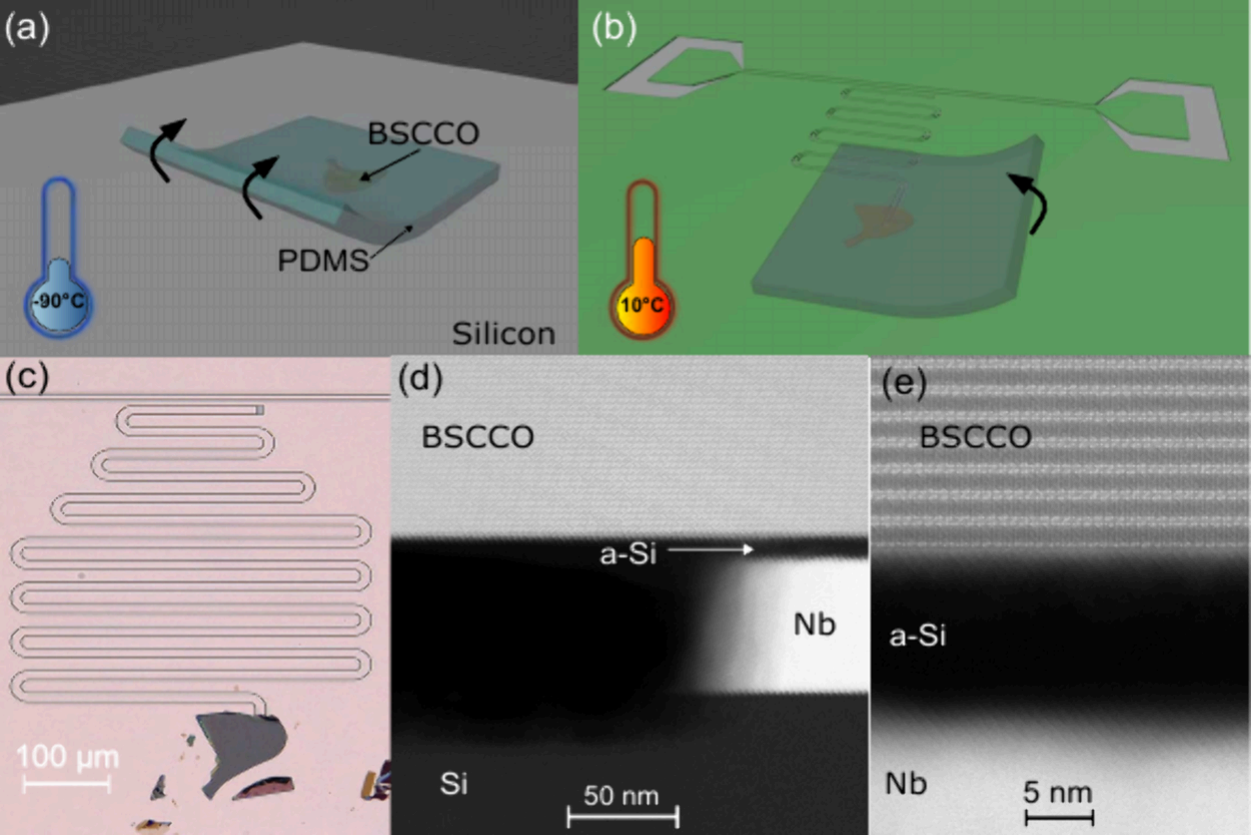
Fabrication and characterization of a hybrid circuit. (a) A BSCCO flake is picked up using a cold PDMS stamp at −90 °C. (b) The flake is placed on top of the niobium resonator, and the temperature is raised to 10 °C to remove the PDMS. (c) Optical image showing the completed hybrid device. (d) Scanning Transmission Electron Microscopy (STEM) image capturing the interface between BSCCO and the niobium at the edge of the central stripe of the resonator. (e) Close-up of the niobium-BSCCO cross-section showing atomically pristine BSCCO layers at the interface.

To quantify the effect of the BSCCO flake on the superconducting resonator, we designed a coplanar waveguide device composed of three different resonator types on the same chip (Figure 2a): a quarter-wave resonator shorted to the ground plane at the end (R1, λ/4), a half-wave resonator with open boundary conditions on both beginning and end (R2, λ/2), and a half-wave resonator which is shorted to ground by a BSCCO flake placed on it (R3), as illustrated in Figure 2b. All three resonators are of 12 μm center line width, 1 μm gap width, and equal lengths, and are coupled to a single 50 Ω transmission line, ensuring consistency in sample quality and minimal lithographic process variations. The scattering parameter across the transmission line was measured while the sample was cooled to 50 mK, revealing resonance peaks corresponding to the three devices, as shown in Figure 2(c-e). The resonance frequency depends on the resonance wavelength λ as *f*_res_ ∝ 1/λ. In this sense, the resonance frequency of the half-wave resonator mode (λ/2 = *l*) is expected to be twice that of the quarter-wave resonator (λ/4 = *l*), consistent with resonators R1 and R2. The resonator R3 with a BSCCO flake shorting the center line to ground resulted in a resonance frequency of 6.28 GHz, a substantial reduction from the half-wave resonance frequency of 10.47 GHz, showing the strong participation of the BSCCO flake in the resonant mode. This intermediate frequency suggests that the resonant mode with BSCCO lies between a λ/4 and λ/2 mode, as depicted in Figure 2f. This mode can be modeled assuming a finite capacitance between the BSCCO flake and the niobium resonator, which was numerically simulated to be approximately 5 pF (see Supporting Information). Concerning the quality factor, both R1 (*Q*_*int*_ = 6 × 10^4^) and R2 (*Q*_*int*_ = 8 × 10^4^) exhibit higher values compared to R3 (*Q*_*int*_ = 3 × 10^4^). The bound on the loss due to hybridization with the flake can be estimated as *Q*_*flake*_ = (1/*Q*_*R*3_ – 1/*Q*_*R*2_)^−1^ = 5 × 10^4^. The discrepancy between the quality factors could possibly be attributed to imperfections in the flake,^46^ but the general high quality of the hybrid device indicates the BSCCO flake in our device maintains good superconducting properties. Additionally, we observed that silicon capping enhances the quality of the hybrid device compared to the resonators without silicon capping (see Supporting Information). This is possibly due to the mitigation of surface defects that degrade the coupling with the BSCCO.^47^

**Figure 2:**
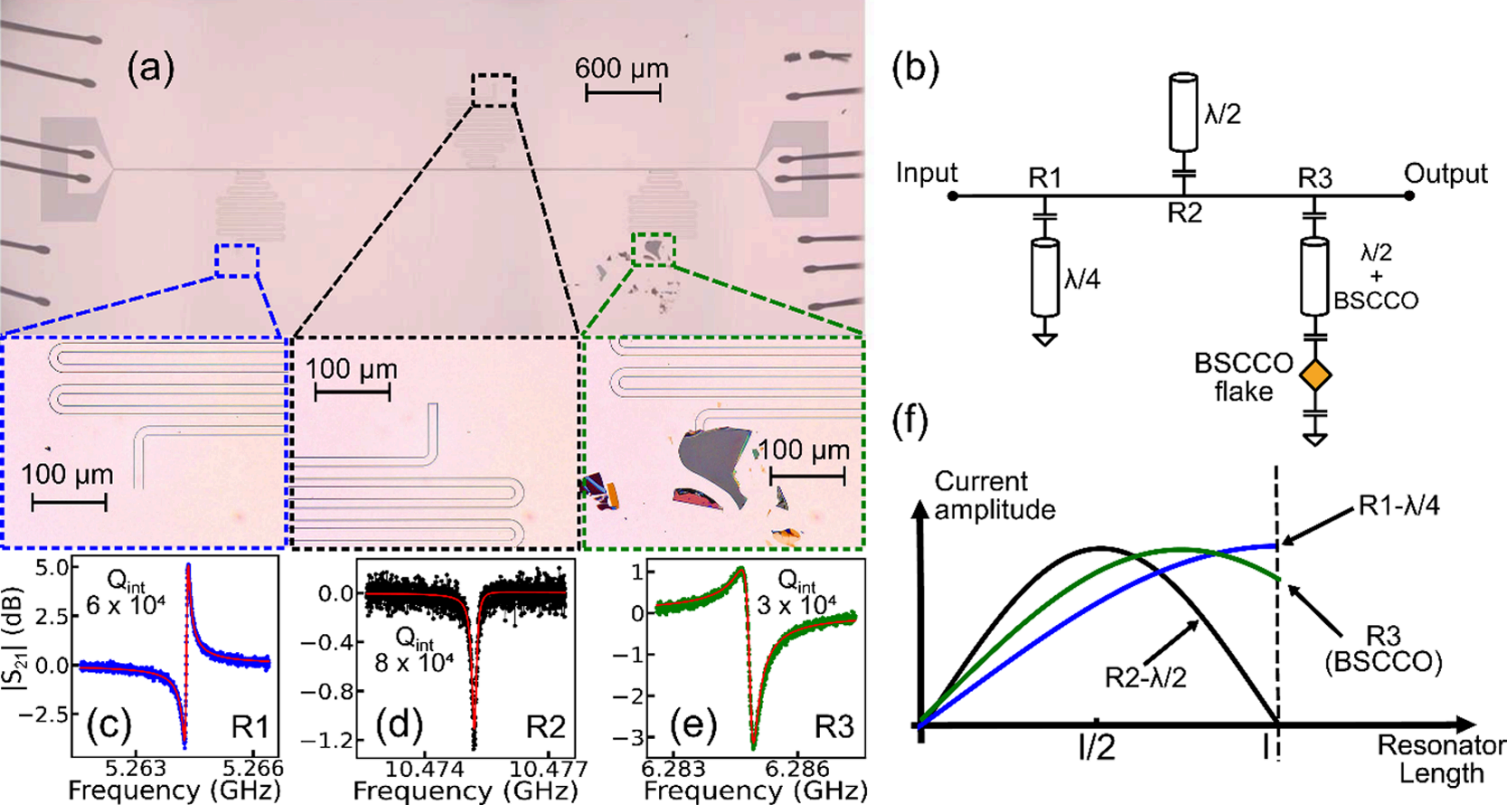
Optical micrography and transmission spectrum. (a) Optical microscope image of a transmission line coupled to three coplanar resonators of equal length but with different boundary conditions: resonator R1 (conventional half-wave, left), R2 (conventional quarter-wave, middle) and R3 (half-wave shunted by a BSCCO flake, right), with enlarged images of the respective resonator terminations. (b) Diagram of the transmission line and resonators. (c-e) Low-power transmission spectrum of the resonator R1 (left, blue), R2 (middle, black) and R3 (with BSCCO flake, right, green) and their internal quality factors at 50 mK. (f) The distribution of current amplitude along the length of the resonator for the different resonant modes.

To explore the microwave properties of the BSCCO flake, we measured the temperature response of the resonance frequency on both the bare λ/2 resonator (R2) and the hybrid λ/2 + BSCCO resonator (R3), as shown in Figure 3. For R2 (Figure 3a), the decrease in the resonance frequency with increased temperature is consistent with the thermal generation of equilibrium quasiparticles in a fully gapped superconductor (*δf*/*f*_0_) ∝ e^–Δ/*T*^^16,48^ with a fitted superconducting gap of Δ_*Nb*_ = 1460 μeV that aligns with previous measurement of thin film niobium.^49,50^

**Figure 3:**
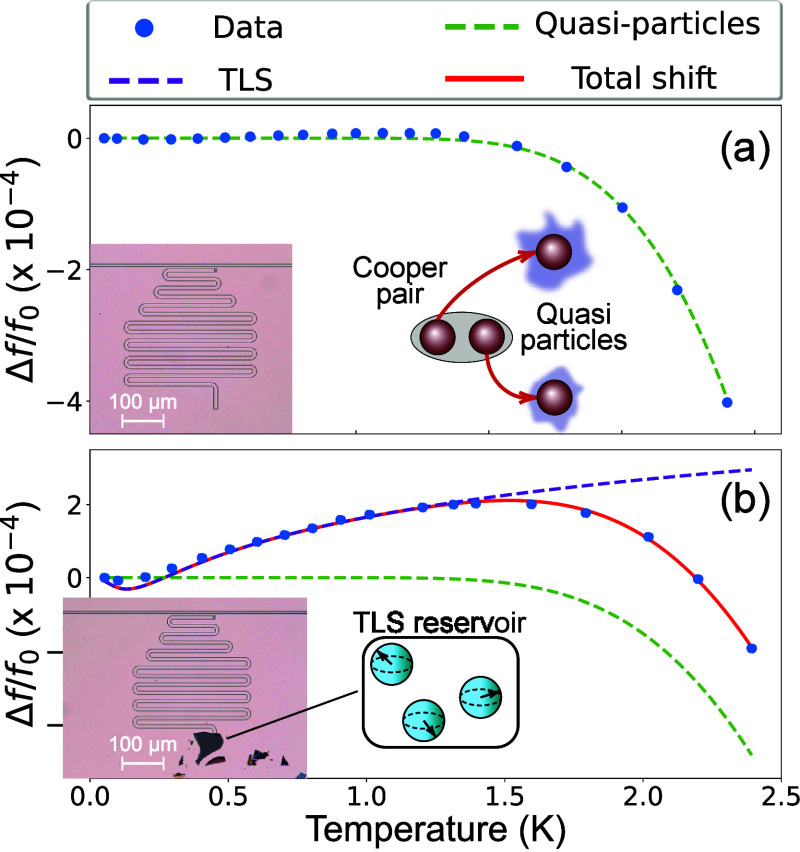
Temperature dependence of the resonance frequency. (a-b) Fractional change of the resonance frequency as a function of temperature for: Resonator R2, λ/2 niobium resonator (a) and resonator R3, λ/2 niobium resonator with a BSCCO flake (b). The green dashed line is a fit to a shift due to the thermal generation of quasiparticles, while the purple dashed line is a fit to coupling with a TLS reservoir. The red line shows a fit to a combined model of both.

On the other hand, the λ/2 resonator with the BSCCO flake (R3) significantly deviates from this conventional behavior (see Figure 3b), showing instead a substantial upshift in the resonance frequency with increasing temperature. This behavior was consistently observed across several different BSCCO hybrid resonators (See Supporting Information Figure S6). In superconducting resonators, a frequency upshift is often attributed to the interaction between the device and a two-level system (TLS) bath.^21,51,52^ The TLS bath is sometimes associated with charged imperfections in the dielectric material.^53^ As the system’s temperature increases the TLS bath saturates, leading to a suppression of the dispersive shift on the device and thus an upshift of the resonance frequency at higher temperature. The interaction with the TLS bath can be modeled by the following equation:^21^
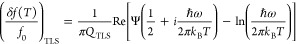
1where Ψ(*x*) is the complex digamma function, and *Q*_TLS_ = 1/*F* tan(δ) is the projected quality factor due to TLS loss which is proportional to the effective coupling *F* and the material loss tangent tan(δ). We fit the resonance frequency shift of the hybrid resonator (R3) with a model that combines the TLS contribution and that of thermal quasiparticles:
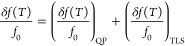
2This model achieves good agreement with the data while introducing only a single additional fit parameter (*Q*_TLS_), and the fitted superconducting gap of the hybrid resonator R3 is consistent with the bare niobium resonators (R1, R2)

However, the quality factor we obtain from the fit model *Q*_TLS_ = 2300 is 1 order of magnitude lower than the total internal quality factor of our device (R3). This discrepancy can be understood by considering a nonuniform spectral distribution of the TLS bath.^54^ While TLS across a broad frequency range can contribute to a frequency shift, dissipation due to the TLS bath is dominated by resonant TLS with a frequency similar to the resonance frequency (*f*_TLS_ ≈ *f*_*r*_). Thus, our data seems to suggest that the TLS bath in the BSCCO flake is mainly composed of off-resonant TLS.

To further investigate the internal mechanisms within the BSCCO flake, we perform measurements on resonators R2 and R3 at varying drive powers. For the resonator R2 without the BSCCO flake, *Q*_*int*_ improves by a factor of 4 with increasing power (see Figure 4a). At low temperatures, the quality factor shows weak temperature dependence, before decreasing rapidly due to the appearance of thermal quasiparticles. This low-temperature power dependence is consistent with dielectric TLS loss in bare superconducting resonators,^55−57^ and matches the slight frequency upshift observed in Figure 3a. For hybrid resonator R3, however, *Q*_*int*_ appears power independent (see Figure 4b), indicating that it is limited by a different mechanism than the TLS in the bare niobium resonator. Additionally, *Q*_*int*_ decreases with temperature with no visible plateau. While this can be caused by various effects, one potential explanation could be excess nodal quasiparticles excited even at low temperatures, due to the d-wave superconducting symmetry of BSCCO.^15,58^

**Figure 4:**
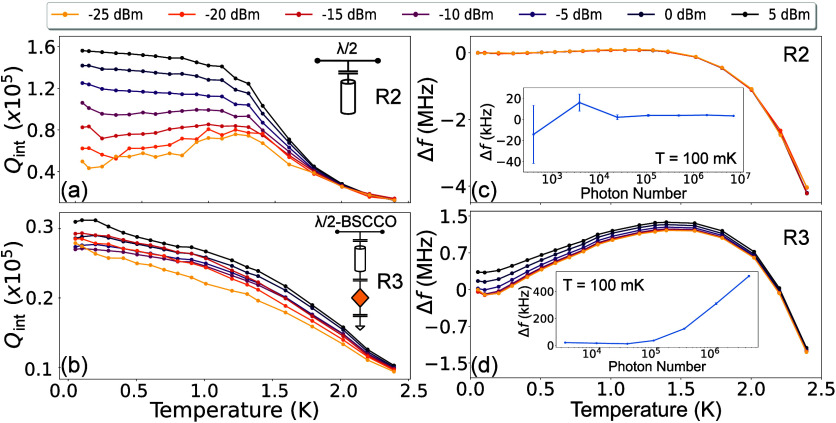
Power dependence of the quality factor and resonance frequency. (a-b) The internal quality factor *Q*_*int*_ vs temperature for different values of applied microwave power for resonator R2 (a) and resonator R3 (b). (c-d) Shift in the resonance frequency vs temperature for different values of applied microwave power for resonator R2 (c) and resonator R3 (d). The inset shows frequency shift as a function of the applied microwave power at 100 mK, expressed in terms of the average intracavity photon number.

The resonance frequency power dependence also shows a distinct behavior for the hybrid circuit. While the bare resonator R2 is power independent within the measured power range, shown in Figure 4c and its insert, the hybrid resonator shows a significant positive frequency shift (Figure 4d) and bifurcation consistent with Kerr nonlinearity. By estimating the number of photons in the resonator, we can obtain an effective Kerr nonlinearity of *K* = 0.1 Hz/photon at 100 mK (See Supporting Information). This value is comparable to those observed in high kinetic inductance resonators,^59,60^ but notably, it is of the opposite sign. As the positive nonlinearity in Josephson devices requires precisely engineered multijunction elements with external flux bias,^61−63^ the nonlinearity in the hybrid device is unlikely to be caused by Josephson or kinetic inductance effects. Note that as the temperature increases, this nonlinearity diminishes, suggesting it is related to the saturation of the TLS bath. However, a TLS bath typically has negligible effect on the power dependence of the resonator frequency.^21,64^ Recently, TLS-induced nonlinearity has been observed in several experiments in which an off-resonant pump tone was added to modify the spectral distribution of TLS defects.^65−67^ Our observation of positive nonlinearity with a resonant frequency drive is thus additional evidence that the TLS bath of the hybrid device has an intrinsic nonuniform spectral density. Another possibility is that the nonlinearity is due to nonequilibrium redistribution of nodal quasiparticles.^68^

Our results show the unique behavior of saturable modes within the flake, but their microscopic origin is currently unknown. One contribution to the overall signal could come from the “pancake” layered vortex structure in BSCCO crystals. Their short coherence length between the CuO_2_ planes results in weak pinning.^69^ Thus, at low temperatures it is possible that a single pancake pinning regime can be detected by the superconducting resonator.^70^ However, given the finite size of the BSCCO flake in comparison to a BSCCO bulk crystal, it is also possible that effects from scale-free and intertwined lattice/charge/spin stripe inhomogeneities^71^ could be detected. Another possible contribution could be due to interaction with mechanical modes in the flake,^46,72,73^ but it is unlikely as similar effects are observed for flakes of significantly different thickness (See Supporting Information Table S1).

In summary, we successfully integrated a BSCCO flake into a niobium coplanar resonator circuit by cryogenic transfer, demonstrating a significant advance in the coupling of high-temperature superconductors with superconducting circuits. The incorporation of BSCCO altered the resonant mode while maintaining a high-quality factor (3 × 10^4^). Temperature-dependent measurements of the hybrid device revealed a significant upshift in the resonance frequency consistent with coupling to a TLS bath. However, the high quality factor of the resonator is inconsistent with strong coupling to such a system, indicating that the TLS bath is mainly off-resonant. The hybrid device also exhibited significant positive nonlinearity, suggesting a new source of lossless nonlinearity unrelated to the Josephson effect. In addition to the novel observations of the BSCCO flake, this work shows a path toward high quality hybrid superconducting circuits with vdW materials and highlights their use in the exploration of unconventional superconductors and the development of new devices for quantum technology applications.^74−76^

## Supporting Information Available

In the Supporting Information, we discuss in more detail the coupling capacitance simulation, the fabrication process of resonators and hybrid devices, the setup for DC and microwave measurements in a dilution fridge, and STEM images of the BSCCO flake-Niobium interface. We also include a comprehensive description of all the data obtained from different devices, with remarks on the nonlinear behavior observed in the hybrid device and the standard TLS effect in bare Niobium resonators. Additionally, we explain the experimental steps taken to exclude effects caused by the cryogenic transfer technique. (PDF)
