# Supplementary material for: Exploring van der Waals Cuprate Superconductors Using a Hybrid Microwave Circuit
Source: Nano Lett. 2025 Jan 27;25(8):3191–8. doi: 10.1021/acs.nanolett.4c05793 (PMC11869361; doi:10.1021/acs.nanolett.4c05793)
Supplement: Supplementary file 1 — nl4c05793_si_001.pdf [file nl4c05793_si_001.pdf]

Supporting Information for
"Exploring van der Waals cuprate superconductors using a hybrid microwave circuit"

Haolin Jin,[1,2,*] Giuseppe Serpico,[1,3,*] Yejin Lee,[1] Tommaso Confalone,[4,5] Christian N. Saggau,[4,6,7] Flavia Lo Sardo,[4,8] Genda Gu,[9] Berit H. Goodge,[1] Edouard Lesne,[1] Domenico Montemurro,[3] Kornelius Nielsch,[4,5,8] Nicola Poccia,[3,4] and Uri Vool[1,4,†]

[1]*Max Planck Institute for Chemical Physics of Solids, 01187 Dresden, Germany*
[2]*Institute of Solid State and Material Physics, Technische Universität Dresden, 01062 Dresden, Germany*
[3]*Department of Physics, University of Naples Federico II, Via Cintia, 80126 Naples, Italy*
[4]*Leibniz Institute for Solid State and Materials Science Dresden (IFW Dresden), 01069 Dresden, Germany*
[5]*Institute of Applied Physics, Technische Universität Dresden, 01062 Dresden, Germany*
[6]*DTU Electro, Department of Electrical and Photonics Engineering, Technical University of Denmark, 2800 Kongens Lyngby, Denmark*
[7]*Center for Silicon Photonics for Optical Communications (SPOC), Technical University of Denmark, 2800 Kongens Lyngby, Denmark*
[8]*Institute of Materials Science, Technische Universität Dresden, 01062 Dresden, Germany*
[9]*Condensed Matter Physics and Materials Science Department, Brookhaven National Laboratory, Upton, NY 11973, USA*

[*] These authors contributed equally to this work.

[†] uri.vool@cpfs.mpg.de

Supporting Information Table of Contents

**A**	**Coupling capacitance simulation**	**2**
**B**	**Fabrication of niobium resonators**	**2**
**C**	**Preparation of the hybrid device**	**3**
**D**	**Measurement Setup**	**4**
**E**	**Scanning Transmission Electron Microscopy images**	**4**
**F**	**Measuring Internal Quality factor Q_{int}**	**5**
**G**	**Microwave response of different hybrid resonators coupled to BSCCO**	**6**
**H**	**Power dependence measurements on the scattering parameters and photon number calibration**	**7**
**I**	**TLS effects in the bare niobium resonator**	**8**
**J**	**Distinguishing the effect of residual PDMS**	**9**
	**References**	**10**

Appendix A: Coupling capacitance simulation

When the BSCCO is coupled to the resonator, it is expected to effectively short the circuit, transitioning its boundary conditions from half wavelength to quarter wavelength. However, experimental measurements reveal a resonance frequency of 6.285 GHz, which deviates from the anticipated value of 5.264 GHz. This discrepancy suggests that the BSCCO flake should not be treated as galvanically coupled to the resonator, and its coupling can be modelled by having a finite capacitance between the resonator and the flake. To investigate this quantitatively, we adopted a circuit model, as illustrated in Figure S1(a), where the BSCCO is treated as an inductance and the coupling to the resonator and ground plane are treated as two equal capacitors C_{BSCCO}. Initially, to estimate the capacitance, we conducted simulations using a circuit simulator (Qucsstudio), performing S-parameter simulations for capacitance values ranging from 100 fF to 10 pF. The results depicted in Figure S1(b) illustrate the variation in resonance frequency with changes in the capacitive coupling of the BSCCO. Around 9 pF, the simulation yields a resonance frequency value consistent with experimental data. Subsequently, a more detailed 3-dimensional S-parameter simulation was performed using HFSS-Ansys (Figure S1(c)). This simulation revealed that the coupling capacitance is approximately 5 pF, providing a more accurate estimation of the BSCCO resonator coupling characteristics.

Appendix B: Fabrication of niobium resonators

The niobium thin films were sputtered on pure intrinsic Si <100> single-crystal substrates using a BESTEC ultra high vacuum magnetron sputtering system. Before deposition, the chamber was evacuated to a base pressure of less than 8×10^{-9} mbar, while the process gas (Ar 5 N) pressure was set to 3×10^{-3} mbar. The target-to-substrate distance was fixed at 20 cm, and the substrate was rotated during the deposition to ensure a homogeneous growth. The sputtering process took 40 mins for a 60 nm thick film. To avoid oxidation of the films, an amorphous Si layer of 6 nm was deposited in situ. The entire process was done at room temperature.

To pattern the resonator, SML300 EBL resist was spun on the film at 2000 rpm for 40 secs with a ram rate of 1000 rpm/sec for an approximate resist thickness of 500 nm and soft baked at 180 °C for 5 minute. The EBL resist was patterned using a VOYAGER (Raith nanofabrication, Germany). The resist was developed in de-ionized water/isopropanol (3/7) solution for 60 s and rinsed in isopropanol for 10 s. After development, the mask was hard baked at 80 °C for 30 minutes. Using the patterned resist as a mask, we etched with the niobium using a dry etch technique. The etching parameters we used were: ambient pressure 0.015 mbar, CHF_3 flow rate 20 sccm, CF_4 flow rate 10 sccm, O_2 flow rate 3 sccm, He flow rate 7 sccm, ICP power of 250 W, and RF bias power of 15 W at a temperature of 30 °C. After etching, the resist mask was removed in Acetone with 10 min sonication followed by rinsing in isopropanol. Finally, O_2 plasma was applied for 10 min to remove the residual polymers.

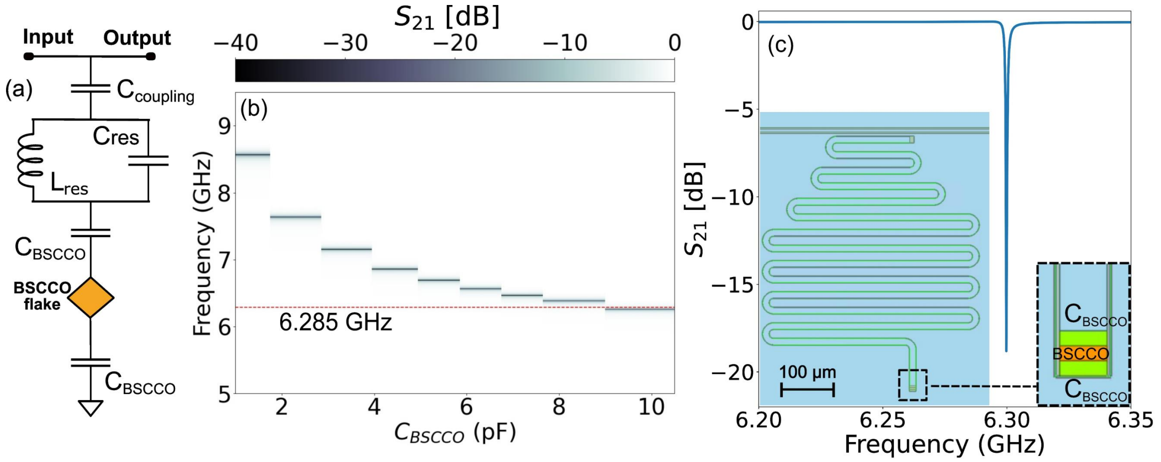

Figure S1. **BSCCO capacitance simulation**. (a) Schematic of the circuit representing a superconducting resonator coupled to a BSCCO flake through C_{BSCCO}. (b) Simulation results of the S_{21} parameters using QUCS for different values of C_{BSCCO}. The red line indicates the experimentally measured value of the resonance frequency. (c) HFSS-Ansys simulation of the circuit, providing a more accurate model of the system. The simulation results suggest a coupling capacitance of approximately 5 pF. The inset shows a view of the coupling region between the resonator and the BSCCO flake.

Appendix C: Preparation of the hybrid device

We have placed the BSCCO flakes on top of the microwave resonator made of niobium using the cryogenic transfer technique in the glovebox (see main text). Right after the sample was taken out of the glovebox, we have wirebonded the sample to a circuit board. Then the device was immediately loaded in a dilution fridge within half an hour. To test the quality of the flake, we have performed the same procedure while transferring a 40 nm-thick BSCCO flake onto gold contacts for transport measurement. Figure S2 shows a superconducting transition at 90 K, comparable to that of a bulk BSCCO crystal. In Ref. [1], BSCCO flakes placed on a contact using the same stacking technique have shown similar results.

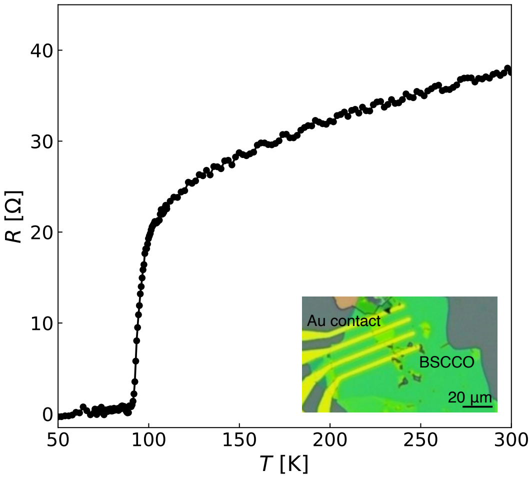

Figure S2. Resistance as a function of temperature of a BSCCO flake that was transferred using PDMS.

Appendix D: Measurement Setup

All devices were measured in a BlueFors LD400 dilution refrigerator with a base temperature of approximately 30 mK measured on the sample holder. A diagram showing the layout for our cryostat and measurement lines is given in Figure S3. All measurements were conducted with a P9373A Keysight Streamline Series USB Vector Network Analyzer.

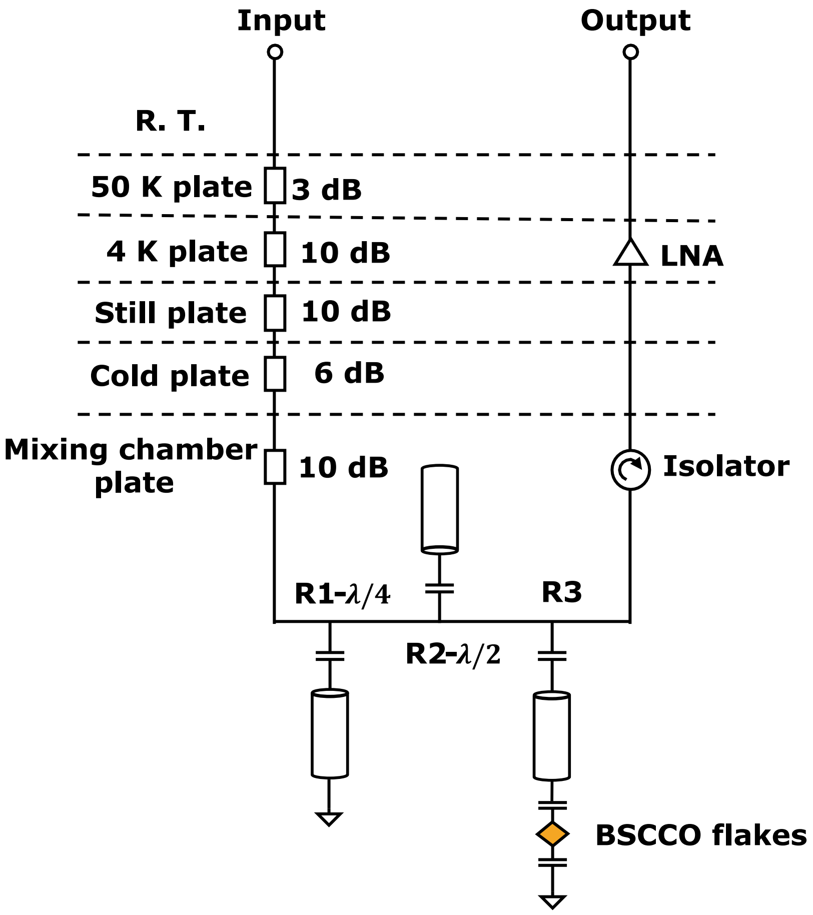

Figure S3. **Diagram of the microwave characterization setup**.

Appendix E: Scanning Transmission Electron Microscopy images

We analyzed the structural details of the resonator coupled to the BSCCO flake by sectioning the hybrid devices and using Scanning Transmission Electron Microscopy (STEM) on the region where the BSCCO contacts the resonator. Figure S4(a) shows a comprehensive STEM image of the resonator coupled to the BSCCO flake, highlighting the overall structure and coupled interface. In the initial devices that we fabricated the BSCCO flake was landed directly on the Niobium resonator. However, as shown in Figure S4(b) and (c), the niobium oxidation resulted in a NbO_x layer, which, due to surface defects, degraded the coupling with BSCCO.

To address this issue, we deposited a 6-nm-thick amorphous silicon capping layer immediately after the niobium deposition, as shown in Figure S5. This modification mitigates the surface defects and enhances the coupling between the niobium resonator and the BSCCO flake, demonstrating an improvement in the device performance.

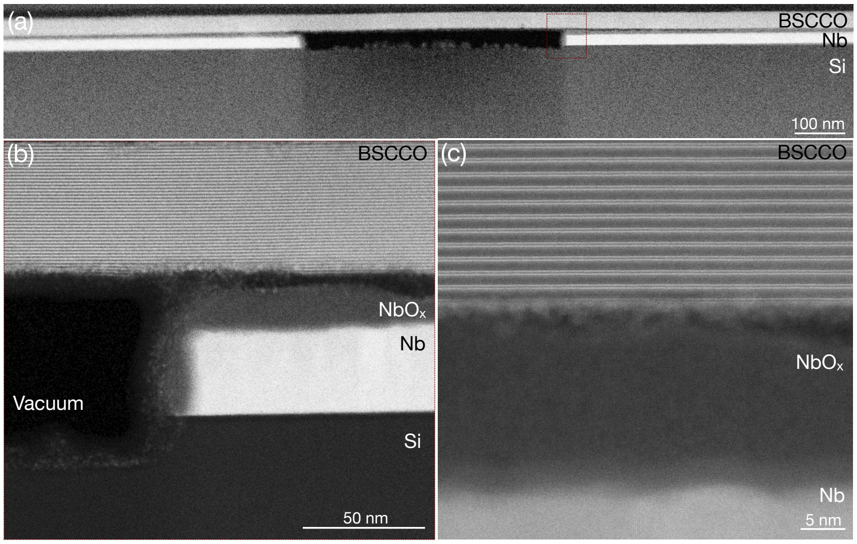

Figure S4. **TEM image of hybrid device coupled to a BSCCO flake without silicon capping**. (a) STEM image of the overall structure of the BSCCO flake coupled to the niobium resonator without a capping layer on the niobium film. (b) Zoomed-in STEM image at the red dashed box in (a). (c) Close up of the interfaces of BiO atomic layers / niobium oxide layer / niobium. Without silicon capping, a rough 15-nm thick niobium oxide layer is formed on the surface of niobium. The BSCCO layers show extensive degradation when in closer contact with the niobium oxide layer.

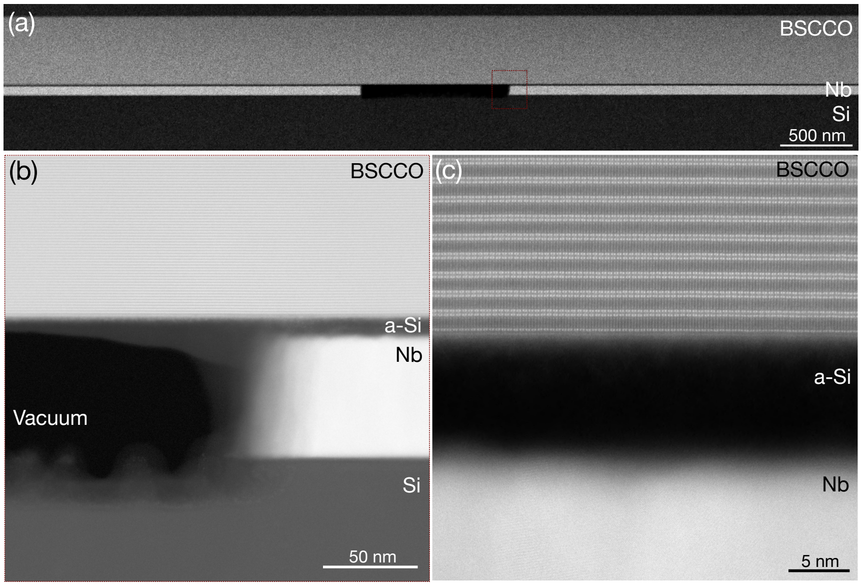

Figure S5. **TEM of hybrid device coupled to a BSCCO flake with silicon capping**. (a) STEM image of the overall structure of the BSCCO flake coupled to the niobium resonator with silicon capping. (b) Zoom-in at the red dashed box of the cross section. (c) Zoom-in of the interfaces of BiO atomic layers of BSCCO flakes/amorphous silicon/niobium, emphasizing the pristine interface between the flakes and resonator. The niobium oxidation was mitigated with a 6-nm-thick amorphous silicon capping layer.

Appendix F: Measuring Internal Quality factor Q_{int}

In this paper, we used a general model to analyze the complex scattering coefficient (S_{21}) of a notch type resonator[2, 3] :

$$S_{21}^{\text{trans}}(f) = \frac{(Q_{tot}/\,|Q_c|)\, e^{i\phi}}{1+2iQ_{tot}\,(f/f_r-1)} \tag{F1}$$

where f denotes the measured frequency, f_r the resonance frequency, Q_{tot} the total quality factor and $|Q_c|$ the absolute value of the coupling quality factor, and ϕ quantifies the impedance mismatch.

Appendix G: Microwave response of different hybrid resonators coupled to BSCCO

In this supplementary section, we present additional devices of hybrid resonators coupled to BSCCO flakes (Figure S6), focusing on the temperature dependence of their resonance frequencies and their internal quality factors at different microwave power levels. For each of the resonators, we observed a shift in the resonance frequency as a function of temperature, which fits well with the hybrid model of quasiparticle excitation and interaction with a TLS reservoir. This behavior aligns with the trends in the hybrid BSCCO device (R3) investigated in the main paper, where the BSCCO flake influences the resonator's performance in a similar manner. We further analyzed the internal quality factor of the resonators at various applied power levels. The additional measurements show that the frequency upshift as well as the positive nonlinearity are robust across multiple devices.

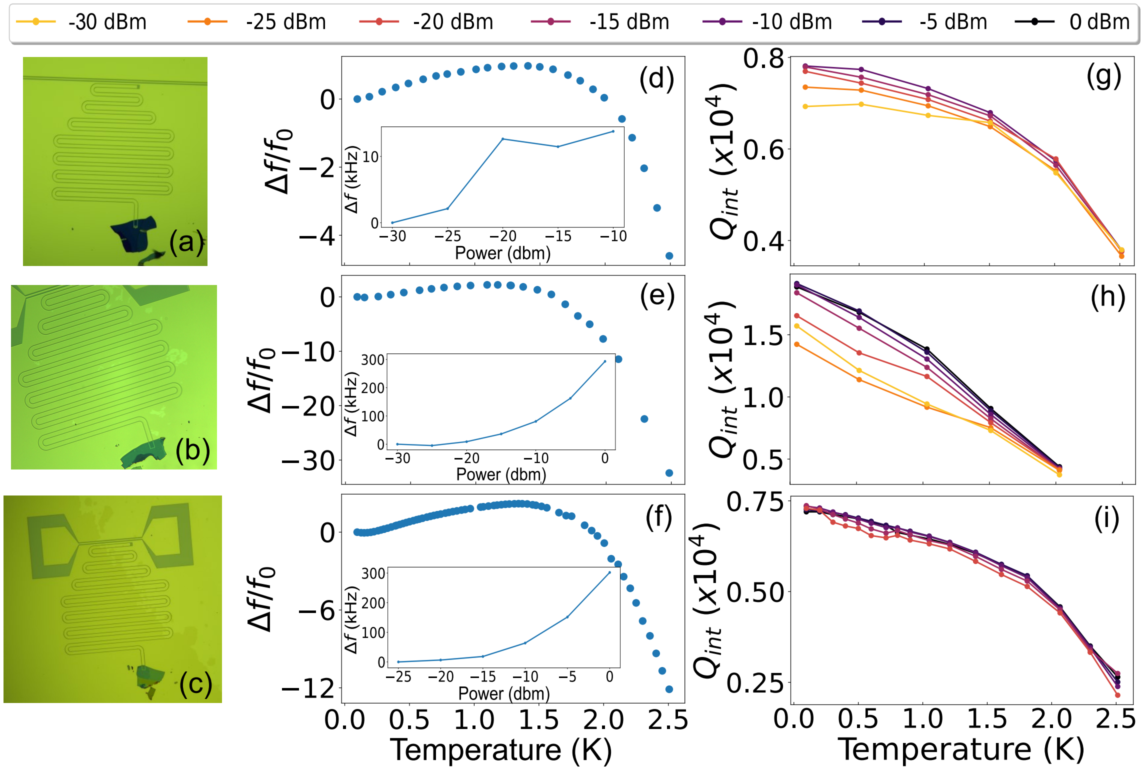

Figure S6. **Microwave response of hybrid BSCCO devices**. (a-c) Optical micrographs of the three resonators coupled to a BSCCO flake. (d-f) Temperature dependence of the resonance frequencies, respectively to the devices in (a-c). (g-i) Analysis of the internal quality factor at different power levels, further confirming the enhanced performance with the silicon capping layer.

Resonator	Internal Quality Factor	Quality Factor of TLS	Thickness of BSCCO flake (nm)	Si Capped
R3	3×10^4	2256	450	Yes
Supp.A	9.1×10^3	4450	68	No
Supp.B	1.9×10^4	1527	55	No
Supp.C	7.6×10^3	1651	83	No

Table S1. Summary of measured devices. The internal quality factor Q_{int}, the quality factor from the fit to the TLS model Q_{TLS}, and the thickness of the flake are shown for hybrid device R3 from the main text and for the three supplemental devices shown in Figure S6.

Table S1 gives a summary of the measurements extracted from the different devices. We observe that the internal quality factor is raised with the introduction of a silicon capping layer. However, the TLS quality factor seems unaffected by the change in the internal quality factor and the presence of a silicon capping layer.

Appendix H: Power dependence measurements on the scattering parameters and photon number calibration

S_{21} measurements of the device R3 at various driving powers are shown in Figure S7. A positive frequency shift with increasing power can be observed, as well as a tilt in the resonance peak. This shift in the resonance shape is observed in the Duffing oscillator and is an indication of nonlinearity and entry into the bifurcation regime.

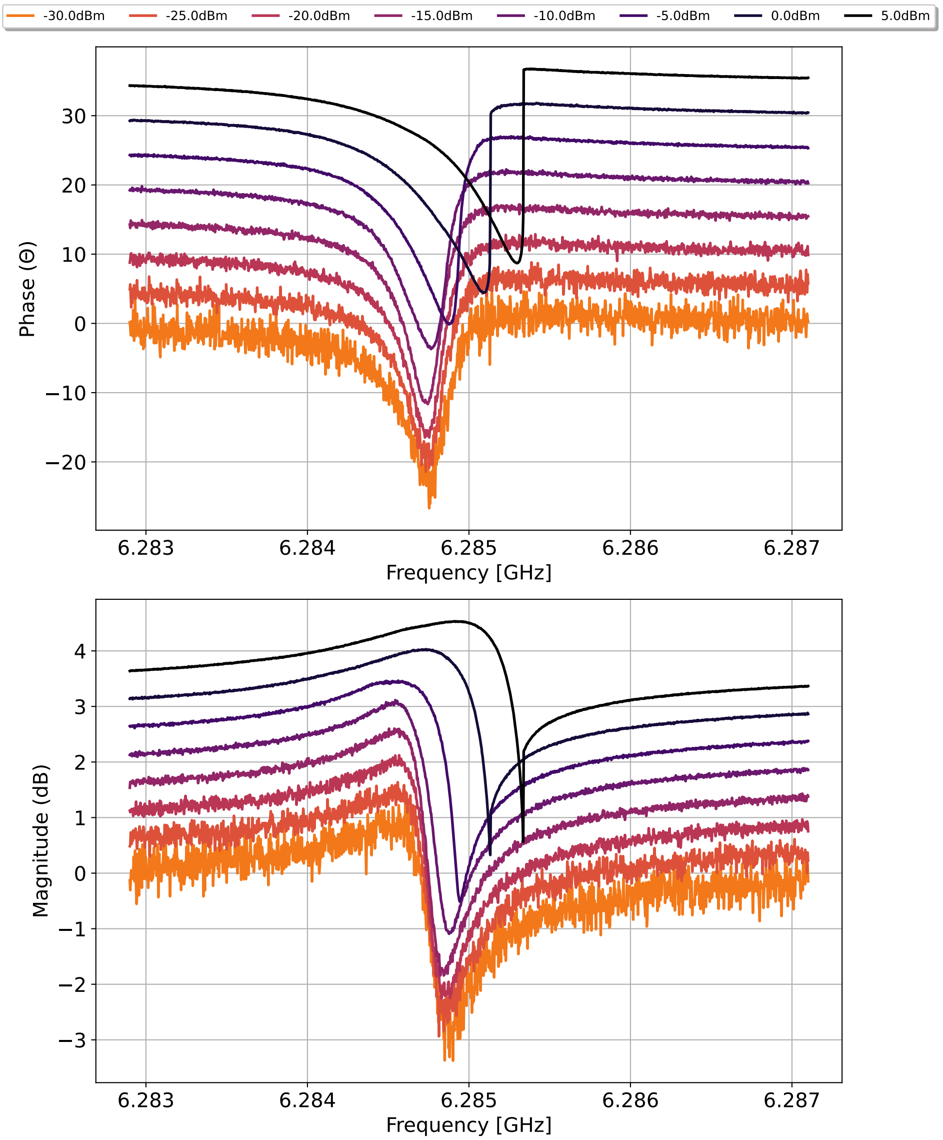

Figure S7. **Nonlinearity of hybrid resonator R3.** The phase and magnitude of the transmission spectrum of the BSCCO flake hybrid resonator for varying inpur powers.

To quantify the nonlinearity in natural units of Hz/photon, we convert the applied driving power into the number of photons in the resonator by using [4]:

$$\bar{N} = P_{in} \frac{4Q_{total}^2}{\hbar\omega_r^2 Q_c} \tag{H1}$$

where $\bar{N}$ is the average photon number inside the resonator, P_{in} is the input power at the transmission line port, Q_{total} is the total quality factor of the resonator, Q_c is the coupling quality factor between the transmission line and resonator and ω_r is the resonance frequency. The largest uncertainty in the photon number estimate comes from the difficulty in accounting for the total

attenuation between the generator and the transmission line and the value of P_{in}. We estimate a total attenuation 80 dB, but the uncertainty in this value indicates that our calibrated photon number should be considered up to one order of magnitude.

Appendix I: TLS effects in the bare niobium resonator

As mentioned in the main text, the bare niobium resonator R2 also shows a slight frequency upshift and its quality factor increases significantly with drive power. Here, we use a combined quasiparticle and TLS model to quantify this effect.

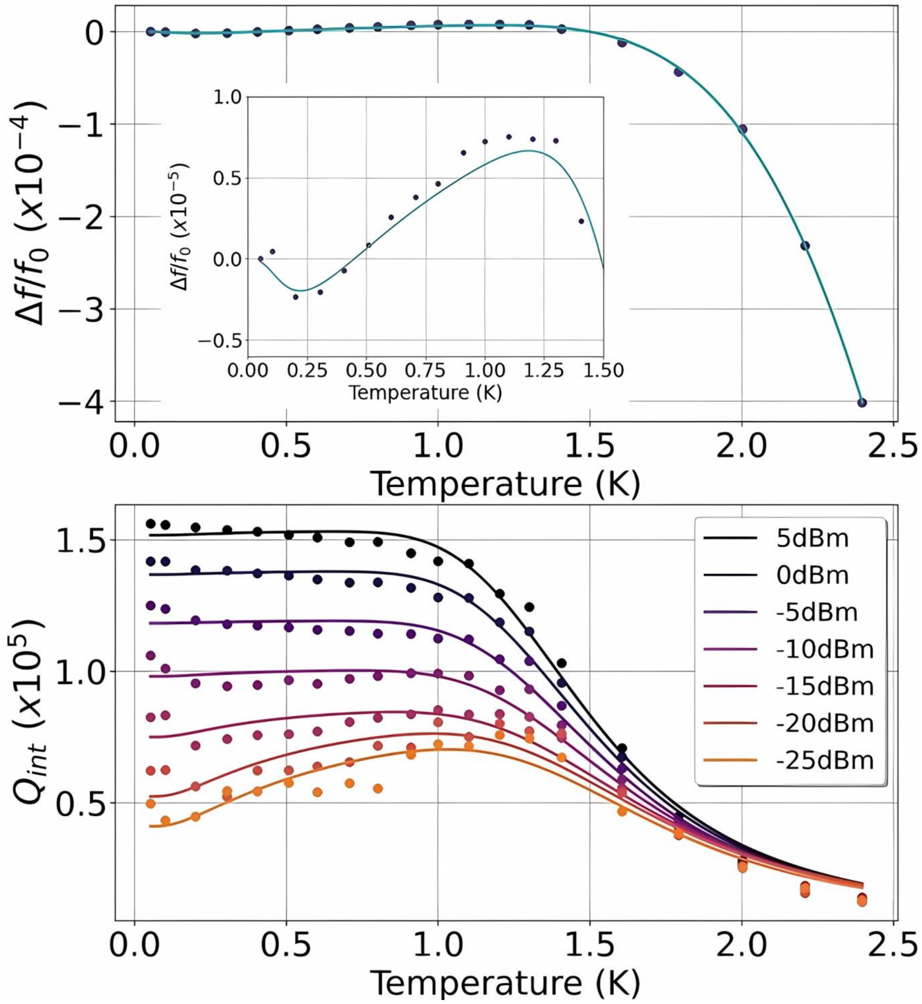

Figure S8. **Parametrizing TLS effects in the bare niobium resonator.** The top panel shows the resonance frequency of resonator R2 vs. temperature, similar to main text Figure 3 but here fit with a combined quasiparticle-TLS model. The inset shows a zoom-in focusing on the frequency upshift at low temperatures. The bottom panel shows the internal quality factor of resonator R2 vs. temperature at different drive powers, fit with a combined quasiparticle-TLS model explained in the text.

In this model, the resonance frequency shift is given by:

$$\frac{\delta f(T)}{f_0} = \left(\frac{\delta f(T)}{f_0}\right)_{\text{TLS}} + \left(\frac{\delta f(T)}{f_0}\right)_{\text{QP}} \tag{I1}$$

where

$$\left(\frac{\delta f(T)}{f_0}\right)_{\text{TLS}} = \frac{1}{\pi Q_{\text{TLS}}} \text{Re}\left[\Psi\left(\frac{1}{2} + i\frac{\hbar\omega}{2\pi k_B T}\right) - \ln\left(\frac{\hbar\omega}{2\pi k_B T}\right)\right] \tag{I2}$$

is the TLS bath contribution to the frequency shift as discussed in the main text [2]. The frequency shift caused by quasiparticles is given by [5]:

$$\left(\frac{\delta f(T)}{f_0}\right)_{\mathrm{QP}} = A\sqrt{\frac{2\pi\Delta_0}{T}}\mathrm{e}^{-\Delta_0/T} \tag{I3}$$

where A is a constant accounting for the participation of the kinetic inductance, and Δ_0 is the superconducting gap. As shown in Figure S8, by fitting the bare niobium resonator with the combined model, we can quantify Q_{TLS} as 3.2×10^4, which is comparable with the internal quality factor of the bare resonator at low drive powers.

Furthermore, we use a combined model to examine the temperature-dependent internal quality factor at different drive powers:

$$\frac{1}{Q_{\mathrm{int}}} = \frac{1}{Q_{\mathrm{TLS}}(T)} + \frac{1}{Q_{\mathrm{QP}}(T)} + \frac{1}{Q_{\mathrm{other}}} \tag{I4}$$

where the $Q_{\mathrm{TLS}}(T)$ is the TLS contribution the quality factor, $Q_{\mathrm{QP}}(T)$ is the quality factor dominated by quasiparticle losses, and Q_{other} accounts for additional temperature-independent losses. The temperature dependence of the quality factors is given by[2, 6]:

$$Q_{\mathrm{TLS}}(T) = Q_{\mathrm{TLS}}/\tanh\left(\frac{\hbar\omega}{2k_B T}\right) \tag{I5}$$

and

$$Q_{\mathrm{QP}}(T) = Q_{\mathrm{QP},0}\frac{e^{\Delta_0/k_B T}}{\sinh\left(\frac{\hbar\omega}{2k_B T}\right)K_0\left(\frac{\hbar\omega}{2k_B T}\right)} \tag{I6}$$

where $K_0(x)$ is the zeroth order modified Bessel function of the second kind.

Figure S8 shows the fit of the bare niobium resonator quality factor to the combined model at various temperatures. The fitted parameter Q_{TLS} increases consistently with the drive power, from 7.2×10^4 at -25 dBm to effectively infinity at the highest driving power, where quasiparticle loss alone can account for the quality factor temperature dependence.

Q_{TLS} extracted from the quality factor measurement is consistent with the one extracted from the resonance frequency. This agreement shows that a broad frequency TLS reservoir accounts for the behavior of the bare niobium resonator, as has been observed in previous resonator studies and attributed to dielectric losses. We note a significant difference between the bare resonator and the hybrid resonator, where the TLS bath shows a large frequency response with minimal effect on the quality factor, possibly due to mostly a reservoir of primarily off-resonant TLS defects. Therefore, it is evident that the source of the anomalous upshift and nonlinearity in the hybrid device is separate from typical TLS effects in superconducting resonators an due to a new mechanism within the BSCCO flake itself.

Appendix J: Distinguishing the effect of residual PDMS

A potential concern of our measurements is that the TLS defects possibly originate from residual PDMS left during the flake transfer procedure. To test this, we have put a PDMS stamp on a niobium resonator without any flake, using the same transfer procedure (see Figure S9a-d). Then we have detached the PDMS stamp and measured the microwave resonance frequency as a function of temperature.

As can be seen in Figure S9e, the resonance frequency gradually drops when the temperature is increased, and appears power independent. This behavior is consistent with a bare resonator and does not show the strong TLS signal we have observed in the hybrid resonators.

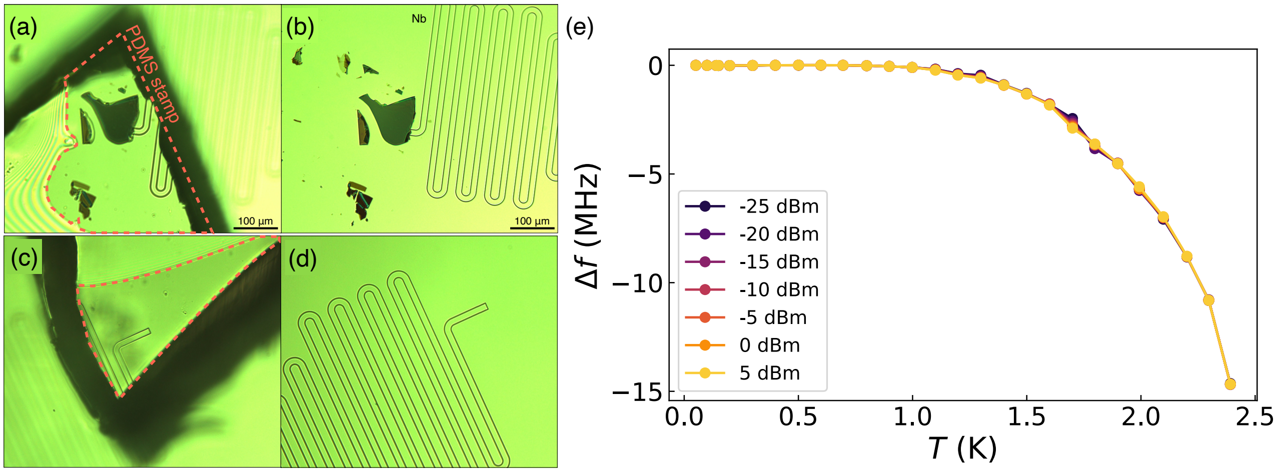

Figure S9. **Control experiment of PDMS** (a-b) Optical micrographs of the transfer process using the PDMS stamp for a flake investigated in the manuscript and the area marked as a red line was in contact with the stamp on the niobium resonator. (c) Optical micrographs of the PDMS stamp attached on the niobium resonator and (d) the niobium resonator after the stamp was detached. (e) The temperature dependent resonance frequency at different applied powers.

[1] Zhao, S., Cui, X., Volkov, P., Yoo, H., Lee, S., Gardener, J., Akey, A., Engelke, R., Ronen, Y., Zhong, R. & Others Time-reversal symmetry breaking superconductivity between twisted cuprate superconductors. *Science*. **382**, 1422-1427 (2023)

[2] Gao, J. The physics of superconducting microwave resonators. California Institute of Technology, 192 (2008)

[3] Probst, S., Song, F., Bushev, P., Ustinov, A. & Weides, M. Efficient and robust analysis of complex scattering data under noise in microwave resonators. *Review Of Scientific Instruments*. **86**, 024706 (2015)

[4] Clerk, A., Devoret, M., Girvin, S., Marquardt, F. & Schoelkopf, R. Introduction to quantum noise, measurement, and amplification. *Reviews Of Modern Physics*. **82**, 1155-1208 (2010)

[5] Prozorov, R. & Giannetta, R. Magnetic penetration depth in unconventional superconductors. *Superconductor Science And Technology*. **19**, R41 (2006)

[6] Mattis, D. & Bardeen, J. Theory of the anomalous skin effect in normal and superconducting metals. *Physical Review*. **111**, 412-417 (1958)
